# Discovery of hub genes linking oxidative stress to type 2 diabetic sarcopenia using single-cell sequencing and machine learning

**DOI:** 10.1371/journal.pone.0352753

**Published:** 2026-07-07

**Authors:** Guangwen Zhu, Kai Zou, Yi Liang, Liting Xie, Qiu Chen

**Affiliations:** 1 Chengdu University of Traditional Chinese Medicine Clinical College, Chengdu, China; 2 Zunyi Traditional Chinese Medicine Hospital, Zunyi, China; 3 Hospital of Chengdu University of Traditional Chinese Medicine, Chengdu, China; Zhejiang University of Technology, CHINA

## Abstract

Type 2 diabetes mellitus (T2DM) and sarcopenia demonstrate a significant comorbidity, particularly in the elderly, yet the molecular mechanisms linking them, especially through oxidative stress, remain incompletely understood. This study aimed to identify oxidative stress-related hub genes involved in T2DM-associated sarcopenia (T2DS) by integrating single-cell RNA sequencing (scRNA-seq) and bulk RNA-seq data with machine learning. We analyzed scRNA-seq datasets (GSE244515, GSE268953) to characterize cellular heterogeneity and bulk RNA-seq datasets (GSE202295, GSE226151) for differential expression. Cell type annotation revealed key involvement of neuromuscular junctions and myofibers. Functional enrichment analyses highlighted pathways like the proteasome, TNF signaling, and ubiquitin-mediated proteolysis. From an initial set of oxidative stress-related genes, a comprehensive machine learning framework comprising 127 algorithm combinations was employed. The Lasso+Stepglm[both] model identified 12 candidate genes. Subsequent Protein-Protein Interaction (PPI) network analysis refined this to seven core hub genes: TNFRSF1B, PSMA2, UBE2D1, UBE2N, HSP90AA1, RAD23A, and DNAJB1. These genes are functionally interconnected, primarily implicating TNFRSF1B-mediated inflammatory signaling that activates the ubiquitin-proteasome system, leading to enhanced protein degradation—a key pathway in muscle atrophy. ROC curve analysis confirmed the strong diagnostic value of these hub genes across training, test, and external validation sets. Our findings systematically reveal novel oxidative stress-related hub genes and mechanisms in T2DS, providing potential biomarkers and therapeutic targets for this debilitating condition.

## 1 Introduction

Diabetes mellitus (DM) is a chronic metabolic disease whose primary pathological changes are absolute or relative insulin deficiency and/or insulin resistance (IR) in target organs. The 8th edition of the International Diabetes Federation Diabetes Atlas (2017) indicated that there are approximately 425 million people with diabetes worldwide, and this number is projected to rise to 700 million by 2045 [1]. The UK Prospective Diabetes Study showed that over 90% of people with diabetes have Type 2 diabetes mellitus (T2DM) [2]. Elderly individuals with T2DM are more prone to comorbidities and various common geriatric syndromes [3], among which sarcopenia has emerged as a novel complication in elderly T2DM patients [4]. The prevalence of sarcopenia is 2–3 times higher in individuals with T2DM compared to those without T2DM [5]. Seok Won Park et al. found that the rate of thigh muscle mass decline in T2DM patients is twice that of healthy individuals [6]. Conversely, individuals aged 70 and above diagnosed with sarcopenia have a 1.5 times higher risk of developing diabetes compared to those without sarcopenia [7]. T2DS is characterized by decreased skeletal muscle mass, diminished muscle strength and function, or loss thereof, increasing the risk of falls, fractures, and other complications. Beyond imposing a heavy burden on patients’ daily lives, it also severely impacts their mental health. Therefore, identifying common target hubs for these two diseases is of great significance for subsequent diagnosis and treatment.

Current research on the pathogenesis of T2DS primarily focuses on several aspects: insulin resistance, inflammatory responses, abnormal skeletal muscle metabolic balance, and oxidative stress [8,9]. Oxidative stress may occur through mechanisms such as lipid metabolism disorders, insulin resistance, increased advanced glycation end products (AGEs), or mitochondrial dysfunction [8]. Some researchers have found [10] that increased oxidative stress is inversely proportional to skeletal muscle mass. The body’s hyperglycemic state can lead to excessive production of superoxide anions through various mechanisms. Glucose uptake enhances NF-κB binding in monocytes, resulting in skeletal muscle damage in experimental animals [11]. With prolonged T2DM duration and advancing age, both the structure and number of mitochondria become abnormal, and their function becomes impaired [12,13]. The reduction in mitochondrial quantity triggers a series of reactions between fatty acids and glucose, leading to increased ROS and oxidative stress, which negatively impacts insulin sensitivity [14]. Therefore, identifying oxidative stress-related targets in T2DS holds significant therapeutic potential. This paves the way for innovative research into targeted drugs to treat this condition.

Bioinformatics has flourished in this era of rapid advancements in high-throughput sequencing technology and significantly enhanced efficiency in genomic and proteomic sequencing. Its computational techniques encompass pattern recognition, data mining, machine learning, method visualization, and more, covering functions such as sequence alignment, gene target prediction, protein structure and function prediction, interaction analysis, drug prediction, and screening. These capabilities enable researchers to achieve their scientific objectives and provide robust support for the flourishing development of the biomedical field [15–17]. The current research paradigm typically involves using bioinformatics to screen potential drug targets for diseases, followed by integrating chemogenomics methods to combine genomic insights of drug targets with the chemical structural aspects of compounds. This approach facilitates the discovery of novel drug targets, particularly enabling the identification of potential targets with 3D structures [18,19]. While traditional drug discovery relies on disease phenotypes, the emerging strategy focuses on identifying underlying disease targets, and this method of drug development is gaining increasing popularity [20]. The discovery of a significant drug target often leads to the invention of hundreds or even thousands of new drugs, which undoubtedly fuels global scientific interest in target research. Studies on T2DM are no exception. By leveraging bioinformatics to identify novel comorbid targets, researchers aim to enhance diagnostic accuracy, optimize treatment outcomes, and alleviate the burden of this disease.

In our current study, we plan to conduct database mining to perform comprehensive analysis of single-cell RNA sequencing data and bulk RNA sequencing data related to type 2 diabetic sarcopenia. The analytical framework will include cell cycle analysis, cell communication analysis, gene set enrichment analysis, and functional enrichment analysis. Subsequently, we will employ machine learning approaches combined with protein-protein interaction network analysis to identify key hub targets. This integrated multi-omics strategy will enable systematic investigation of the molecular mechanisms underlying type 2 diabetic sarcopenia, with particular focus on oxidative stress-related pathways and cellular interactions within the neuromuscular microenvironment.

## 2 Materials and methods

### 2.1 Data acquisition

Bulk RNA sequencing (Bulk RNA-seq) data related to type 2 diabetes and sarcopenia were obtained from the Gene Expression Omnibus (GEO) database. Specifically, dataset GSE202295 pertained to type 2 diabetes, while GSE226151 served as the sarcopenia dataset (Table 1). In single-cell RNA sequencing (scRNA-seq), the type 2 diabetes data were derived from two samples (GSM7818495, normal sample; GSM7818516, disease sample) in the GSE244515 dataset; the sarcopenia data were obtained from two samples (GSM8303169, normal sample; GSM8303179, disease sample) in the GSE268953 dataset (Table 2). Furthermore, 14,130 oxidative stress-related genes were initially retrieved from the GeneCards database (https://www.genecards.org/) using the keyword ‘Oxidative stress.’ These genes were subsequently filtered using the Gene Inferred Function Score (GIFtS), a GeneCards metric that integrates multiple evidence sources (e.g., publications, functional assays, protein domains) to assess the likelihood of a gene playing a functional role. A threshold of GIFtS ≥ 10 was applied as it is a commonly used cutoff to retain genes supported by substantial functional evidence while eliminating poorly characterized entries (Threshold screening instructions on Genecard official website). This filtering yielded 11,931 high-confidence oxidative stress-related genes for downstream analysis (S1 Table).

**Table 1:** Bulk RNA-seq.

Dataset	Platform	Data category	Species	Disease	Total quantity	Control quantity	Disease quantity
GSE202295	GPL16791	Bulk RNA-seq	Human	Type 2 Diabetes	111	61	50
GSE226151	GPL16791	Bulk RNA-seq	Human	Sarcopenia	40	20	20

**Table 2:** scRNA-seq.

Dataset	Platform	Data category	Species	Disease	Normal sample	Disease sample
GSE244515	GPL16791	scRNA-seq	Human	Type 2 Diabetes	GSM7818495	GSM7818516
GSE268953	GPL24676	scRNA-seq	Human	Sarcopenia	GSM8303169	GSM8303179

### 2.2 Analysis of scRNA-seq datasets

The R packages (e.g., Seurat, tidyverse, patchwork, harmony, ggplot2, and cowplot) were employed in the analytical workflow. This workflow included quality control, dimensionality reduction, clustering, and annotation steps to investigate cellular heterogeneity and the underlying pathogenesis of T2DS using scRNA-seq data. A detailed description of the analytical steps is provided below.

#### 2.2.1 Quality control.

Low nFeature_RNA values may indicate dead cells or empty droplets, while excessively high nFeature_RNA and nCount_RNA (the total number of molecules detected per cell) could suggest the presence of doublets or multiple cells within a single droplet. The Seurat R package was used to perform quality control on the raw data to filter high-quality cells. The filtering criteria included retaining cells where the number of detected genes per cell ranged from 500 to 5000, and the percentage of mitochondrial and erythrocyte-related gene expression was below 15%.

#### 2.2.2 Dimensionality reduction, clustering, and annotation.

Dimensionality reduction was performed using Principal Component Analysis (PCA). t-Distributed Stochastic Neighbor Embedding (t-SNE) plots were utilized to reduce data complexity, enabling the visualization and clustering of cells in a low-dimensional space. The ElbowPlot() function was employed to help determine the number of significant principal components (PCs) to be used in subsequent analyses. Cell clustering was conducted using the FindClusters function from the Seurat package, with the clustering resolution parameter specified. To further visualize the spatial distribution characteristics of the cell populations, the Uniform Manifold Approximation and Projection (UMAP) algorithm was applied for dimensionality reduction and visualization of the clustering results. Subsequently, each cell cluster was systematically annotated based on known canonical marker genes, enabling precise identification and classification of cell types. Finally, gene sets for each cell type were compiled.

#### 2.2.3 Cell cycle analysis and cell communication analysis.

Cell cycle analysis provides insights into the molecular mechanisms underlying cell proliferation and differentiation. Using the TriCycle package, cell cycle analysis was performed by assigning cells to specific phases (G1, S, and G2/M) based on the Z-scores of cell cycle marker genes, thereby revealing the distribution characteristics of cells across different cycle stages.

In CellChat analysis, permutation tests were used to determine the significance of each ligand-receptor (L-R) pair. Specifically, cell group labels were shuffled, and communication strength was recalculated to generate a null distribution. If the actual communication strength was significantly greater than the random distribution (P < 0.05), the edge was retained. Thus, each edge in the figure represents significant evidence of communication. A small P-value in the output indicates significant communication between cell group A and cell group B. Larger nodes indicate larger cell populations, and thicker edges represent a greater number of interactions. This result reflects the global network of the number of significant communications between cell groups under specific conditions.

### 2.3 Bulk RNA-seq data normalization and gene set enrichment analysis

Transcriptome data normalization is a critical step in Bulk RNA-seq data analysis, aimed at eliminating the effects of irrelevant factors such as sequencing depth and gene length, thereby ensuring comparability of gene expression levels both between and within samples.

Gene Set Enrichment Analysis (GSEA) was employed to evaluate whether a predefined gene set shows statistically significant, concordant differences between two biological states. In this study, the R package clusterProfiler was used to perform enrichment analysis on all genes ranked by logFC between healthy and disease groups in type 2 diabetes and sarcopenia. Gene sets related to muscle fibers and neuromuscular junctions were obtained from the scRNA-seq datasets. Significantly enriched gene sets were identified using a screening threshold of P < 0.05. Subsequently, the intersection between the enriched muscle fiber and neuromuscular junction genes and oxidative stress-related genes was identified. These intersecting genes were considered as potential oxidative stress-related genes associated with T2DS.

### 2.4 Functional enrichment analysis

Disease Ontology (DO) enrichment analysis is a method used to identify and interpret enrichment patterns of disease associations within a given gene list. By mapping a set of genes to disease entries in the DO database, this approach evaluates the significance of gene enrichment in specific disease categories. Gene Ontology (GO) analysis is a widely used method for large-scale functional enrichment studies, covering three domains: biological process (BP), molecular function (MF), and cellular component (CC). The Kyoto Encyclopedia of Genes and Genomes (KEGG) is a comprehensive database that integrates genomic, pathway, disease, and drug information.

In this study, the R package *clusterProfiler* was employed to perform DO, GO, and KEGG enrichment analyses on oxidative stress-related genes enriched in muscle fibers and neuromuscular junctions. A significance threshold of *P* < 0.05 was applied as the screening criterion.

### 2.5 Identification of hub genes using machine learning models

To identify oxidative stress-related hub genes in T2DS, the GSE202295 dataset was randomly divided into training and test sets via stratified sampling at a 7:3 ratio. All original expression data were standardized using the scale function prior to modeling (mean = 0, standard deviation = 1) to eliminate the influence of dimensional differences among different samples or features. After retaining only common genes, the training set and test set were standardized independently, respectively. To enhance the generalization and robustness of the models, minor Gaussian noise (mean = 0, standard deviation = 0.01) was added to the training set and test set separately during the analysis process.

A two-stage feature selection strategy was adopted. First, for each machine learning method with feature selection capability (Lasso, SVM, RF, glmBoost, Stepglm, Ridge, Enet, GBM, LDA, XGBoost, and NaiveBayes), the algorithm was run independently on the training set to automatically screen significant variables; the minimum threshold for the number of variables was set to 2. If the number of variables after screening was insufficient, variables were supplemented to meet the threshold based on the P-values of the t-test. All variable screening methods uniformly called the custom RunML function, and parameter optimization was performed using 10-fold cross-validation (e.g., Lasso/Enet/GBM).

Based on the aforementioned feature sets, each algorithm was used to construct the final model separately. Common methods included Lasso regression (glmnet, alpha = 1), Ridge regression (glmnet, alpha = 0), Elastic Net (glmnet, alpha ranging from 0 to 1), Random Forest (randomForestSRC, ntree = 1000, nodesize = 5), GBM (gbm, n.trees = 10000, interaction.depth = 3, shrinkage = 0.001, cv.folds = 10), Support Vector Machine (e1071), Naive Bayes (e1071), Partial Least Squares Generalized Linear Model (plsRglm), BART, etc. For combined methods (e.g., “Lasso+SVM,” “glmBoost+Ridge”), features were first screened by the former method, followed by modeling with the latter. Some methods (e.g., stepwise regression) adopted stepwise screening (parameters set as “forward,” “backward,” or “both”).

After model training, external validation was performed using the test set. Classification prediction output risk scores (probabilities) and category labels, with the area under the curve (AUC) as the evaluation metric, calculated using the pROC package. Finally, all methods were ranked by the mean AUC. The final model selection is based on three criteria: high AUC value, low risk of overfitting, and gene set size ≤ 15. Ultimately, ROC curves were plotted using the pROC package in R software, and the AUC values of the training set, test set, and external validation cohort were calculated to analyze and evaluate the diagnostic value of the hub genes.

### 2.6 Construction of protein-protein interaction (PPI) network

Hub targets were imported into the STRING database with the species set to “Homo sapiens” and the interaction confidence score threshold set to 0.4. Combined with the visualization of the Protein-Protein Interaction (PPI) network diagram, in-depth analysis of network topological parameters was performed to screen out core targets. Cytoscape software was used to visualize the core targets based on the PPI network diagram. Subsequently, the Uniform Manifold Approximation and Projection (UMAP) algorithm was applied to examine the expression patterns of the 7 targets in type 2 diabetes and sarcopenia. To verify the accuracy of the hub targets, differential expression analysis was conducted using scRNA-seq datasets of sarcopenia and type 2 diabetes. Finally, KEGG pathway enrichment analysis was re-performed to identify the signaling pathways activated by the hub genes.

## 3. Results

### 3.1 Analysis of scRNA-seq datasets

#### 3.1.1 Quality control.

**Fig 1:**
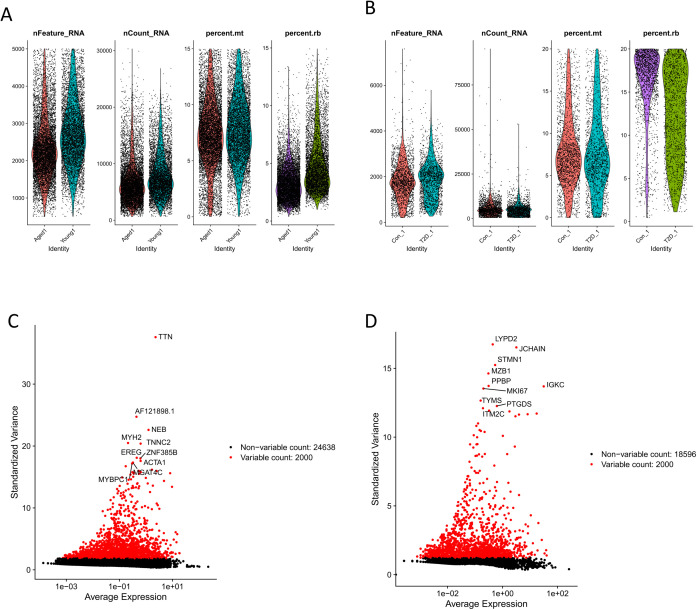
Quality Control. A. Sarcopenia: Violin plots post-quality control. B. Type 2 Diabetes: Violin plots post-quality control. C. Sarcopenia: Significant volcano plot. D. Type 2 Diabetes: Significant volcano plot.

Quality control was performed on datasets for type 2 diabetes and sarcopenia. Following quality control procedures (Figs 1A and B), the distribution of feature counts became more tightly clustered, indicating improved consistency in molecular characteristics of the cell populations after removal of low-quality cellular data. Genes with high significance were subsequently visualized (Figs 1C and D). Results revealed that genes significantly associated with sarcopenia included TTN (titin), ACTA1 (actin alpha 1), MYBPC1 (myosin-binding protein C1), all of which are closely related to muscular function. In contrast, the gene LYPD2, implicated in insulin secretion and glucose metabolism, was identified as significantly associated with type 2 diabetes.

#### 3.1.2 Dimensionality reduction, clustering, and annotation.

**Fig 2:**
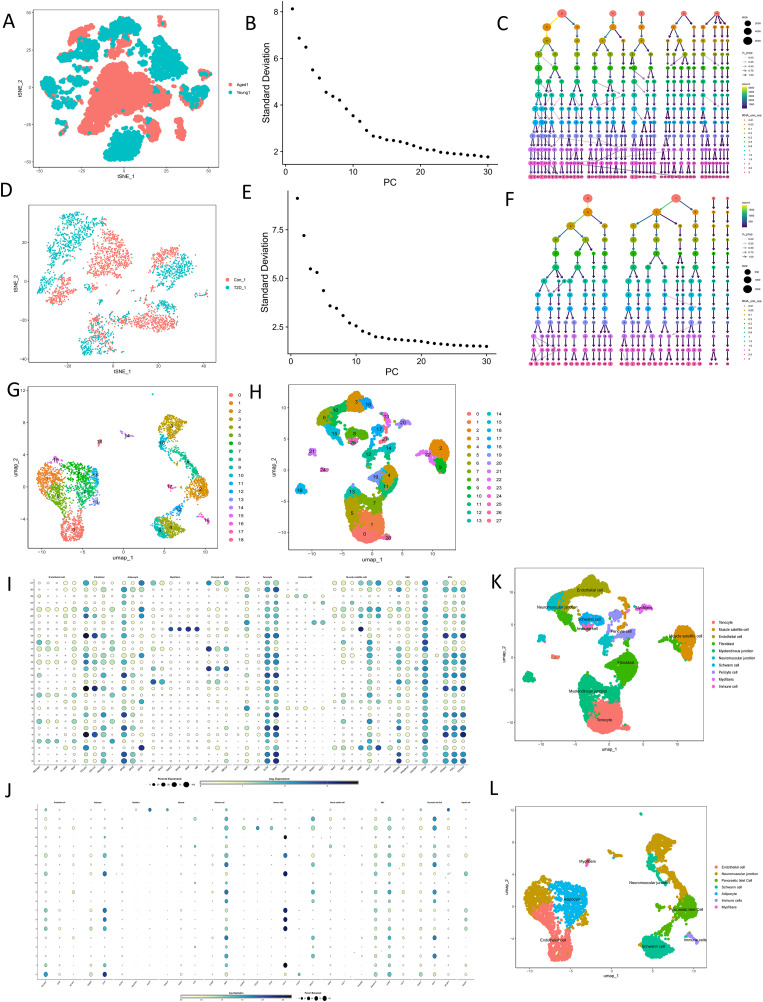
Dimensionality Reduction, Clustering, and Annotation. A. Data dimensionality reduction for sarcopenia (disease vs. control groups). B. Number of principal components for sarcopenia. C. Resolution parameter for sarcopenia. D. Data dimensionality reduction for type 2 diabetes (disease vs. control groups). E. Number of principal components for type 2 diabetes. F. Resolution parameter for type 2 diabetes. G. Cell clusters in sarcopenia. H. Cell clusters in type 2 diabetes. I. Canonical marker genes for sarcopenia. J. Canonical marker genes for type 2 diabetes. K. Cell type annotation for sarcopenia. L. Cell type annotation for type 2 diabetes.

Dimensionality reduction via Principal Component Analysis (PCA) was performed for both diseases, with the top 20 principal components (PCs) extracted. t-SNE was subsequently applied to reduce data complexity, enabling the visualization and clustering of cells in a low-dimensional space (Figs 2A and D). The results demonstrated clear separation between the disease and control groups.

The ElbowPlot function was used to determine the number of significant PCs for subsequent analysis. Cell clustering was conducted using the FindClusters function from the Seurat package, with the clustering resolution parameter specified. For sarcopenia, the number of PCs was set to 14 and the clustering resolution parameter was set to 1.2 (Figs 2B and C); for type 2 diabetes, the number of PCs was set to 12 and the clustering resolution parameter was set to 1.5 (Figs 2E and F). To further visualize the spatial distribution of cell populations, the Uniform Manifold Approximation and Projection (UMAP) algorithm was applied for dimensionality reduction and visualization of the clustering results (Figs 2G and H). The results showed that cells were divided into 27 distinct clusters in sarcopenia and 18 clusters in type 2 diabetes.

Subsequently, based on relevant studies and canonical marker genes from CellMarker 2.0 (http://117.50.127.228/CellMarker/), the biological types of each cell cluster were manually annotated (Figs 2I and J, Tables 3 and 4), each cell cluster was manually annotated to achieve precise identification and classification of cell types (Figs 2K and L). The results indicated that cell types in sarcopenia included tenocytes, muscle satellite cells, endothelial cells, fibroblasts, myotendinous junctions, neuromuscular junctions, Schwann cells, pericytes, myofibers, and immune cells. In type 2 diabetes, identified cell types comprised endothelial cells, neuromuscular junctions, islet cells, Schwann cells, adipocytes, immune cells, and myofibers. Based on literature review, it is recognized that the comorbidity of T2DS is associated with neuromuscular junctions and myofibers [21]. therefore, subsequent analyses will focus on these two cell types.

**Table 3:** Manual Annotation Table of Single-Cell Data for Sarcopenia.

Cell Type	Associated Markers
Endothelial cell	PECAM1, CDH5, KDR, NCAM1, ENG
Fibroblast	COL3A1, COL1A1, PDGFRA, ITGA6
Adipocyte	APOD, GPX3, LEP, APOE
Myofibers	ACTA2, SMA, COL1A1, PDGFRA, MYLK, TNNT3, MYH2, CKM, MB
Pericyte cell	RGS5, ACTA2, NOTCH3, ABCC9
Schwann cell	PLP1, MBP
Tenocyte	COL1A1, SCXA, FMOD, DLG2, FBN1
Immune cells	FCER1G, CD14, C1QA, SKAP1, IKZF1
Muscle satellite cell	PAX7, MET, CD82, ASB5, CAV1, DLK1
NMJ	CHRNA1, PHLDB2, PRKAR1A, COL25A1, UTRN
MTJ	COL22A1, COL6A1, FSTL1, COL6A3

**Table 4:** Manual Annotation Table of Single-Cell Data for Type 2 Diabetes.

Cell Type	Associated Markers
Endothelial cell	PECAM1, CDH5, ENG, NCAM1
Adipocyte	APOD, ADIPOQ, CEBPA, CFD, CCL21
Myofibers	ACTA2, PDGFRA, MYLK
Myocyte	ACTA1, TNNC2, TNNI1, TNNI2, MYL1, TTN
Schwann cell	CDH19, PLP1, S100B, MBP, SOX10
Immune cells	CENPE, TOP2A, TPX2, CD14, C1QA, SKAP1
Muscle satellite cell	PAX7, MET, CD82, ASB5, CAV1, DLK1
NMJ	PHLDB2, PRKAR1A, UTRN, VAV3
Pancreatic Islet Cell	INSIG1, CD81, MCUR1
Hepatic cell	MCUR1, ASGR1

#### 3.1.3 Cell cycle analysis and cell communication analysis.

**Fig 3:**
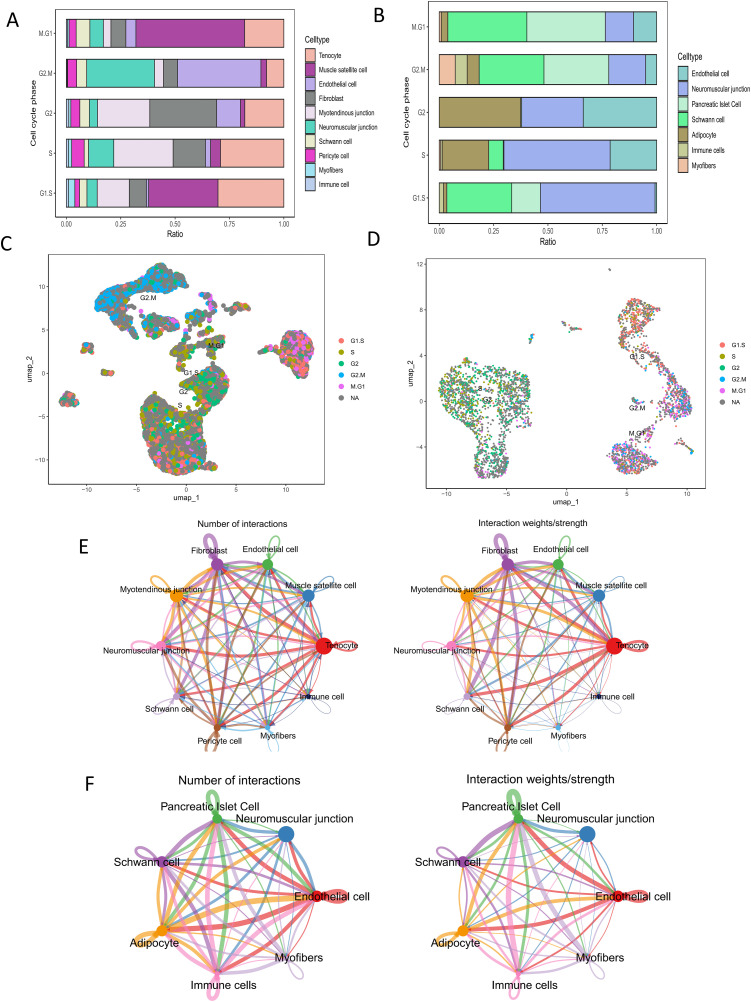
Cell Cycle Analysis and Cell Communication Analysis. A. Sarcopenia: Cell cycle distribution ratio plot. B. Sarcopenia: Cell cycle UMAP plot. C. Type 2 Diabetes: Cell cycle distribution ratio plot. D. Type 2 Diabetes: Cell cycle UMAP plot. E. Sarcopenia: Cell communication network. F. Type 2 Diabetes: Cell communication network.

The TriCycle package was employed to assign cells to specific cell cycle phases (G1, S, and G2/M) based on Z-scores of cell cycle marker genes (Figs 3A-D). Results revealed that in sarcopenia, neuromuscular junction cells showed a slight increase in the G2/M and S phases, while myofibers exhibited a relatively uniform distribution across different cell cycle phases. In type 2 diabetes, neuromuscular junction cells displayed the highest proportions in the G2/M and G1/S phases, while myofibers also maintained a relatively even distribution across cell cycle stages.

Cell communication networks were constructed and analyzed using ligand-receptor interaction information from the CellChatDB database, including interaction strength and weights (Figs 3E and F). Results demonstrated that in both sarcopenia and type 2 diabetes, neuromuscular junctions showed relatively high network interaction strength and interaction weights. Furthermore, myofibers were found to exhibit a certain level of interaction with neuromuscular junctions.

### 3.2 Bulk RNA-seq data normalization and gene set enrichment analysis

**Fig 4:**
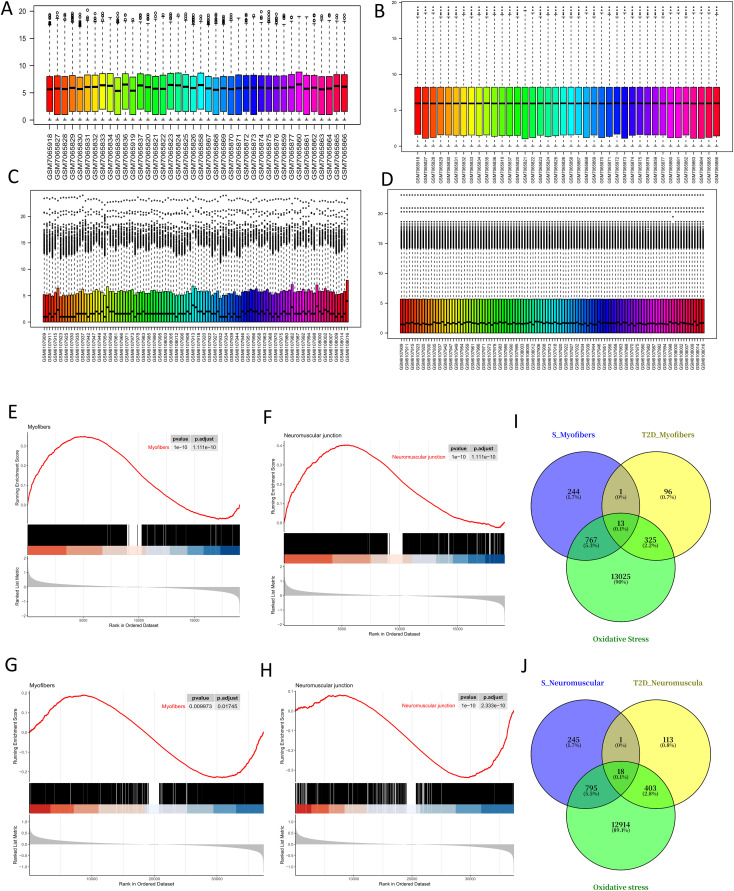
Bulk RNA-seq Data Normalization and Gene Set Enrichment Analysis. A-D. GSE226151 before and after normalization. E-F. Enrichment of sarcopenia in muscle fibers and neuromuscular junctions. G-H. Enrichment of type 2 diabetes in muscle fibers and neuromuscular junctions. I-J. Overlapping genes of oxidative stress in muscle fibers and neuromuscular junctions between sarcopenia and type 2 diabetes.

After data normalization (Figs 4A–4D), box plot analysis revealed a significantly reduced data range and more concentrated distribution, indicating that the data cleaning process likely removed outliers. This enables comparability of gene expression levels across and within samples.

This study performed GSEA to investigate the enrichment of type 2 diabetes and sarcopenia in muscle fibers and neuromuscular junctions (Tables 5 and 6). The results showed that sarcopenia was positively associated with both gene sets (Figs 4E and 4F). In contrast, type 2 diabetes was negatively associated with the neuromuscular junction gene set (Fig 4G), while the number of up-regulated and down-regulated genes enriched in the muscle fiber gene set showed little difference (Fig 4H). Subsequently, the overlapping genes related to oxidative stress in muscle fibers and neuromuscular junctions between the two diseases were identified (Figs 4I and 4J). The results indicated 13 genes associated with oxidative stress in muscle fibers of T2DS, and 18 genes associated with oxidative stress in neuromuscular junctions of T2DS.

**Table 5:** GSEA Analysis of Sarcopenia.

ID	enrichmentScore	NES	pvalue	p.adjust	qvalue	leading_edge	core_enrichment
Schwann cell	2743	0.419183022497951	2.08790995188725	1e-10	1.11111111111111e-10	5130	tags=43%, list=27%, signal=37%
Neuromuscular junction	2228	0.403746266375041	1.98728157157853	1e-10	1.11111111111111e-10	5577	tags=48%, list=29%, signal=38%
Pericyte cell	2027	0.402958538852726	1.9736633210654	1e-10	1.11111111111111e-10	5060	tags=42%, list=26%, signal=35%
Endothelial cell	2514	0.392053234128471	1.94364650289957	1e-10	1.11111111111111e-10	5119	tags=41%, list=27%, signal=35%
Immune cell	2019	0.388057001576761	1.89946193729615	1e-10	1.11111111111111e-10	4801	tags=39%, list=25%, signal=33%
Tenocyte	2368	0.373327485660603	1.84422238100733	1e-10	1.11111111111111e-10	5111	tags=42%, list=27%, signal=35%
Myotendinous junction	2631	0.366318439598036	1.81939923214556	1e-10	1.11111111111111e-10	5515	tags=44%, list=29%, signal=36%
Myofibers	2499	0.348846207982977	1.72828669704365	1e-10	1.11111111111111e-10	5119	tags=41%, list=27%, signal=35%
Muscle satellite cell	3433	0.330994196783658	1.65816733039807	1e-10	1.11111111111111e-10	5577	tags=41%, list=29%, signal=35%
Fibroblast	1817	0.285407133794919	1.39197280059945	2.38556571762801e-08	2.38556571762801e-08	4566	tags=32%, list=24%, signal=27%

**Table 6:** GSEA Analysis of Type 2 Diabetes.

ID	enrichmentScore	NES	pvalue	p.adjust	qvalue	leading_edge	core_enrichment
Immune cells	3962	−0.374010203	−1.850115957	1e-10	2.33333333333333e-10	8880	tags=41%, list=24%, signal=35%
Neuromuscular junction	1516	−0.338758211	−1.612094504	1e-10	2.33333333333333e-10	7842	tags=35%, list=21%, signal=29%
Pancreatic Islet Cell	1810	−0.303127127	−1.446778729	1e-10	2.33333333333333e-10	7930	tags=33%, list=21%, signal=28%
Myofibers	1840	−0.230088686	−1.099108351	0.00997324490647968	0.0174531785863394	6564	tags=24%, list=18%, signal=21%
Endothelial cell	1763	0.257289887127208	1.13393333988819	0.0344062153163152	0.0481687014428413	7928	tags=30%, list=21%, signal=25%
Schwann cell	1658	−0.210362551	−0.998289983	0.475247524752475	0.554455445544555	7933	tags=27%, list=21%, signal=22%
Adipocyte	1735	0.217039308167114	0.956162565538078	0.712374581939799	0.712374581939799	8886	tags=31%, list=24%, signal=25%

### 3.3 Functional enrichment analysis

**Fig 5:**
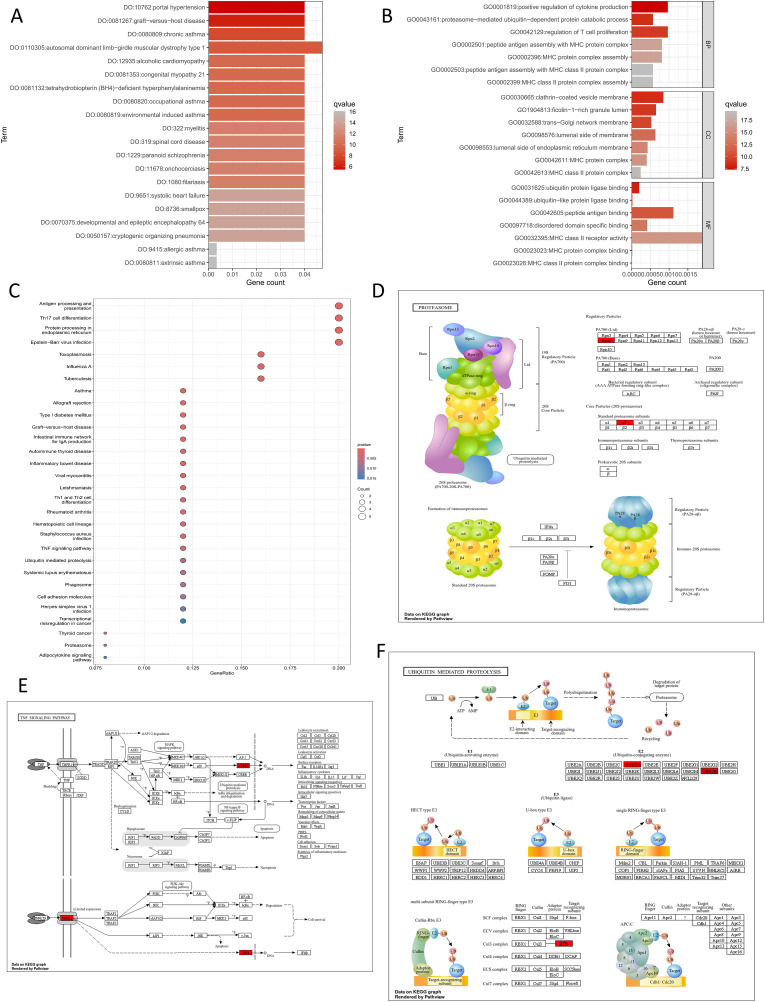
Functional Enrichment Analysis. A. DO enrichment. B. GO enrichment analysis. C. KEGG enrichment analysis. D. Proteasome pathway. E. TNF signaling pathway. F. Ubiquitin-mediated proteolysis pathway.

Functional enrichment analysis was performed on the aforementioned intersecting genes. Disease Ontology (DO) enrichment analysis (Fig 5A) identified 23 significant terms (P < 0.05), primarily related to muscular dystrophy, limb-girdle, type 1 and congenital myopathy.

In Gene Ontology (GO) enrichment analysis (Fig 5B): Biological Process (BP) terms were mainly associated with the positive regulation of cytokine production, proteasome-mediated ubiquitin-dependent protein catabolic process, and assembly of peptide antigens with MHC protein complex. Cellular Component (CC) terms primarily involved MHC class II protein complex and lumen of filamin-1 enriched granules. Molecular Function (MF) terms were chiefly related to ubiquitin protein ligase binding.

Kyoto Encyclopedia of Genes and Genomes (KEGG) enrichment analysis (Fig 5C) revealed significant involvement in pathways including the Proteasome (Fig 5D), TNF signaling pathway (Figure 5E), and Ubiquitin mediated proteolysis (Fig 5F).

### 3.4 Identification of Hub genes using machine learning models

**Fig 6:**
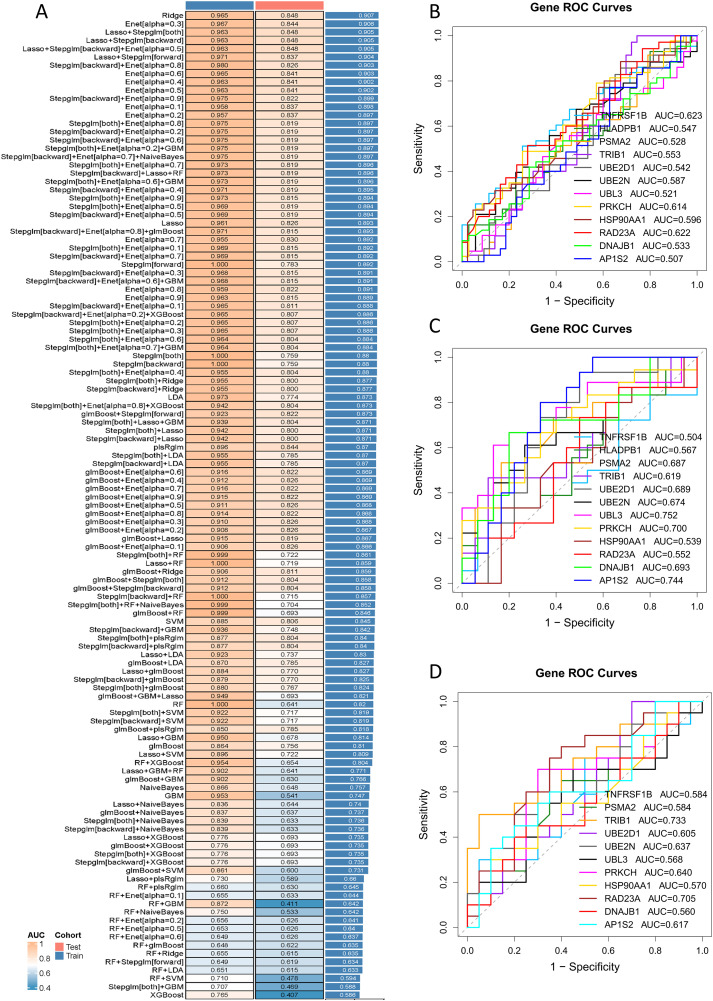
Identification of Hub Genes Using Machine Learning Models. A. Construction of 127 predictive models. B-D. AUC score evaluation in training, testing, and external validation sets.

To identify hub genes associated with oxidative stress in T2DS, the GSE202295 dataset was randomly divided into training and test sets via stratified sampling at a 7:3 ratio, with GSE226151 serving as an external validation cohort. Subsequently, we constructed 127 predictive models to further identify oxidative stress-related genes associated with type 2 diabetes sarcopenia and determine candidate core hub genes. Finally, all 127 models were ranked using the mean AUC. Some top-ranked models achieved good apparent performance primarily by relying on high training-set AUC, but the difference between training and test AUC was generally large, indicating a significant overfitting problem (Fig 6A). Model selection followed three predefined criteria: (i) a high level of AUC value; (ii) consistency of AUC between training and test sets (ΔAUC < 0.1) to minimize overfitting; and (iii) a parsimonious gene set (≤15 genes) to ensure biological interpretability. Consequently, top-ranked models that contained an excessive number of genes (S2 Table) and exhibited large AUC differences between datasets, suggesting overfitting, were excluded. The selected glmBoost+Enet (alpha = 0.6) model was evaluated by ROC curve analysis, demonstrating AUC values greater than 0.5 in the training set, test set, and external validation cohort (Figs 6B-D), indicating that these core genes have significant diagnostic value. Moreover, the training-test AUC difference for this model was only 0.094, indicating a very low risk of overfitting (Fig S1A), while the AUC in the independent test cohort reached 0.822, maintaining excellent discriminative performance with ensured stable generalizability. Additionally, through its regularization mechanism, this model eliminated genes without predictive value (Fig S1B), substantially reducing the number of features while still maintaining a high test-set AUC, balancing model parsimony with clinical generalizability.

In summary, the glmBoost+Enet (alpha = 0.6) model satisfied all three criteria—high AUC value, low overfitting risk, and gene set parsimony—and was therefore selected as the final predictive model. The 12 genes identified by this model were: TNFRSF1B, HLADPB1, PSMA2, TRIB1, UBE2D1, UBE2N, UBL3, PRKCH, HSP90AA1, RAD23A, DNAJB1, and AP1S2.

### 3.5 Protein-protein interaction (PPI) network construction and molecular mechanisms

**Fig 7:**
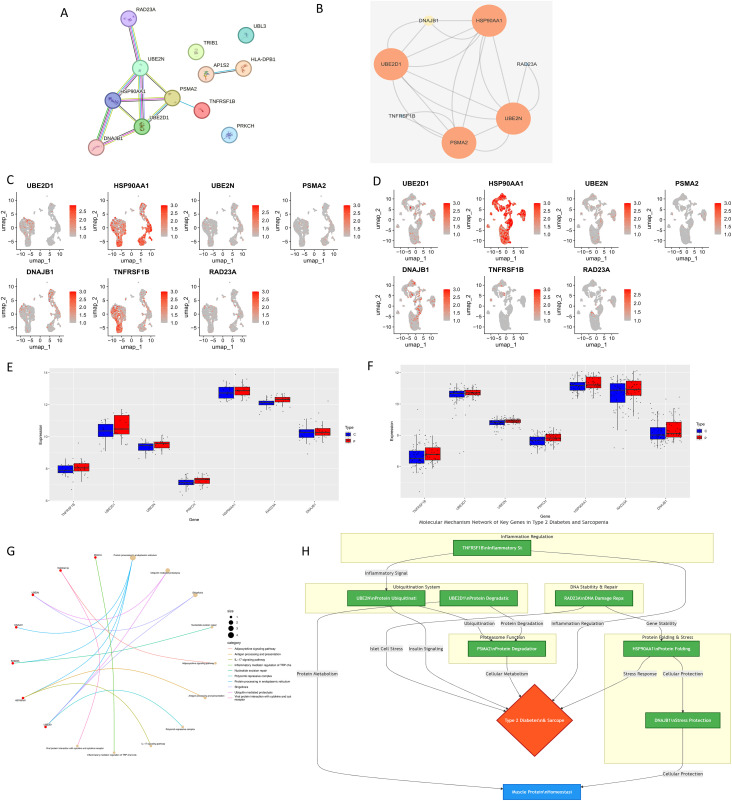
Protein-Protein Interaction (PPI) Network Construction and Molecular Mechanisms. A. PPI interaction network of 12 hub genes. B. PPI interaction network of 7 core hub genes. C. Expression patterns of 7 genes in sarcopenia. D. Expression patterns of 7 genes in type 2 diabetes. E. Box plots of differential expression analysis for the 7 genes in sarcopenia. F. Box plots of differential expression analysis for the 7 genes in type 2 diabetes. G. KEGG enrichment plot for the 7 targets. H. Mechanistic network diagram.

The 12 target genes were imported into the STRING database (Fig 7A, Table 7), revealing interaction relationships among 7 genes, which were identified as the core hub genes. These core targets were visualized using Cytoscape software in conjunction with the PPI network (Fig 7B). Uniform Manifold Approximation and Projection (UMAP) was subsequently employed to examine the expression patterns of these 7 targets in type 2 diabetes and sarcopenia. The results demonstrated that in sarcopenia, TNFRSF1B was primarily expressed in neuromuscular junctions and endothelial cells, while UBE2D1 was expressed across all cell types but showed higher expression in Schwann cells (Fig 7C). In type 2 diabetes, TNFRSF1B was ubiquitously expressed but showed elevated levels in endothelial cells and neuromuscular junctions, whereas UBE2D1 was mainly expressed in neuromuscular junctions and adipocytes (Fig 7D). To further verify the accuracy of the hub genes, differential expression analysis was performed using scRNA-seq datasets of sarcopenia and type 2 diabetes. The results showed that the expression levels of these 7 genes were higher in the disease groups than in the normal groups, both in sarcopenia and type 2 diabetes (Figs 7E and F). Furthermore, KEGG pathway enrichment analysis revealed that the top 2 enriched pathways for the 7 targets were primarily “Protein processing in endoplasmic reticulum” and “Ubiquitin mediated proteolysis” (Fig 7G, Table 8). The mechanism involving these 7 core targets primarily involves TNFRSF1B-mediated inflammatory signaling, which activates the ubiquitination system and subsequently promotes protein degradation (Fig 7H).

**Table 7:** PPI Scores of the 12 Genes.

node1	node2	neighborhood_on_chromosome	gene_fusion	phylogenetic_cooccurrence	homology	coexpression	experimentally_determined_interaction	database_annotated	automated_textmining	combined_score
AP1S2	HLA-DPB1	0	0	0	0	0.084	0	0.5	0	0.522
DNAJB1	UBE2D1	0	0	0	0	0.08	0.046	0	0.586	0.605
DNAJB1	HSP90AA1	0.042	0	0	0	0.374	0.133	0.5	0.999	0.999
HLA-DPB1	AP1S2	0	0	0	0	0.084	0	0.5	0	0.522
HSP90AA1	PSMA2	0	0	0	0	0.393	0.091	0	0.236	0.541
HSP90AA1	DNAJB1	0.042	0	0	0	0.374	0.133	0.5	0.999	0.999
HSP90AA1	UBE2N	0	0	0	0	0.2	0.292	0.5	0.293	0.773
HSP90AA1	UBE2D1	0	0	0	0	0.134	0	0	0.364	0.426
PSMA2	TNFRSF1B	0	0	0	0	0	0	0.4	0	0.4
PSMA2	UBE2D1	0	0	0	0	0.074	0	0.4	0.193	0.513
PSMA2	HSP90AA1	0	0	0	0	0.393	0.091	0	0.236	0.541
PSMA2	UBE2N	0	0	0	0	0.475	0	0	0.189	0.557
RAD23A	UBE2N	0	0	0	0	0.068	0.172	0.5	0.365	0.722
TNFRSF1B	PSMA2	0	0	0	0	0	0	0.4	0	0.4
UBE2D1	PSMA2	0	0	0	0	0.074	0	0.4	0.193	0.513
UBE2D1	DNAJB1	0	0	0	0	0.08	0.046	0	0.586	0.605
UBE2D1	UBE2N	0	0	0	0.918	0.052	0.098	0.5	0.107	0.567
UBE2D1	HSP90AA1	0	0	0	0	0.134	0	0	0.364	0.426
UBE2N	PSMA2	0	0	0	0	0.475	0	0	0.189	0.557
UBE2N	UBE2D1	0	0	0	0.918	0.052	0.098	0.5	0.107	0.567
UBE2N	RAD23A	0	0	0	0	0.068	0.172	0.5	0.365	0.722
UBE2N	HSP90AA1	0	0	0	0	0.2	0.292	0.5	0.293	0.773

**Table 8:** KEGG Analysis of the 7 Targets (Top 5).

ID	Description	GeneRatio	BgRatio	pvalue	p.adjust	qvalue	geneID	Count	Enrichment.Score	Fold.Enrichment
hsa04141	Protein processing in endoplasmic reticulum	4/7	171/9396	3.55269E-06	9.94754E-05	8.97522E-05	UBE2D1/HSP90AA1/RAD23A/DNAJB1	4	5.449442385	31.39849624
hsa04120	Ubiquitin mediated proteolysis	2/7	142/9396	0.004531645	0.063443035	0.057241836	UBE2D1/UBE2N	2	2.343744088	18.9054326
hsa05131	Shigellosis	2/7	253/9396	0.013868994	0.129443941	0.116791526	UBE2D1/UBE2N	2	1.857955049	10.61095426
hsa03420	Nucleotide excision repair	1/7	63/9396	0.046015653	0.1665888	0.150305684	RAD23A	1	1.337094412	21.30612245
hsa04920	Adipocytokine signaling pathway	1/7	70/9396	0.051014597	0.1665888	0.150305684	TNFRSF1B	1	1.292305537	19.1755102
hsa04612	Antigen processing and presentation	1/7	81/9396	0.058824716	0.1665888	0.150305684	HSP90AA1	1	1.230440158	16.57142857

## 4. Discussion

Sarcopenia is one of the comorbidities of T2DM, with a particularly high prevalence among the elderly population [22]. Aging is accompanied by a significant decline in both skeletal muscle mass and function [23–25]. Sarcopenia has become a serious threat to the quality of life in older adults, as affected individuals often face higher risks of falls, fractures, and premature mortality [26–28]. Notably, T2DS interact bidirectionally, forming a positive feedback loop that exacerbates both conditions. The underlying mechanisms involve insulin resistance, vitamin D deficiency, among other factors [8,29,30]. Our study has made preliminary progress by identifying seven core targets associated with T2DS through machine learning and protein-protein interaction network analysis: TNFRSF1B, PSMA2, UBE2D1, UBE2N, HSP90AA1, RAD23A, and DNAJB1. These seven targets demonstrate strong functional correlations. Their primary mechanism involves TNFRSF1B-mediated inflammatory signaling that activates the ubiquitination system, leading to enhanced protein degradation. Significantly, this mechanism aligns with one of the established pathogenic pathways of sarcopenia [31].

Skeletal muscle atrophy is driven by interrelated pathophysiological processes in which oxidative stress and inflammation act as key initiating factors, subsequently activating downstream pathways including the ubiquitin-proteasome system [32]. Inflammatory responses are prevalent in various disease states, particularly in chronic metabolic diseases. Persistent inflammatory stimuli can alter the microenvironment of skeletal muscle cells. T2DS is a typical example of such diseases, where inflammatory signals disrupt the balance between protein synthesis and degradation in skeletal muscle, leading to the occurrence of skeletal muscle atrophy [33]. It is noteworthy that inflammatory stimuli and oxidative stress are inextricably linked, interacting in the pathogenesis of T2DS to form a vicious cycle [34–36]. While prior studies have separately documented the roles of inflammation [31], oxidative stress [10], and the ubiquitin-proteasome system [32] in T2DM or sarcopenia, our work systematically integrates these elements into a unified molecular network centered on seven interconnected hub genes. This integration addresses a critical knowledge gap by revealing how T2DM-specific metabolic stress and sarcopenia-related muscle dysfunction are mechanistically linked at the molecular level.

TNFRSF1B (also known as TNFR2) is a receptor for the pro-inflammatory cytokine TNF-α, with well-established roles in immunoregulation and inflammatory responses [37]. Previous studies have shown that TNFRSF1B expression is upregulated in the adipose tissue and skeletal muscle of T2DM patients, contributing to insulin resistance [37]. In sarcopenia research, the TNF-α/TNFR pathway has been linked to muscle atrophy, but most studies have focused on TNFR1 rather than TNFR2 [31]. Within the inflammatory pathway, heat shock protein 90 alpha (HSP90AA1) also plays a significant role, with studies indicating its ability to induce muscle atrophy by regulating the AKT-mTOR and NF-κB signaling pathways [38]. Ubiquitin-conjugating enzyme E2 N (UBE2N) activates downstream inflammatory pathways by catalyzing the formation of K63-linked polyubiquitin chains, acting as a key amplifier of pathways such as NF-κB and resulting in sustained, low-grade chronic inflammation. This is not only a core link in inducing insulin resistance and muscle wasting but also serves as a critical bridge connecting inflammation and the ubiquitin-proteasome system [39,40]. Our study provides novel evidence that TNFRSF1B serves as the central upstream hub in T2DS, mediating crosstalk between T2DM-associated chronic inflammation and sarcopenic muscle protein degradation. Through PPI network analysis, we demonstrated that TNFRSF1B amplifies inflammatory signaling while also directly interacting with downstream ubiquitin-proteasome components such as UBE2N and HSP90AA1. This dual role bridges two pathogenic processes previously considered disconnected in T2DM and sarcopenia. This dual role of TNFRSF1B in T2DS has not been reported in prior studies, highlighting its potential as a therapeutic target for breaking the comorbidity cycle.

Within the protein degradation pathway, persistent hyperglycemia and an oxidative stress environment lead to extensive damage to muscle proteins and protein misfolding. Under these conditions, the expression of DnaJ heat shock protein family (Hsp40) member B1 (DNAJB1) is upregulated to recognize damaged proteins, and its function shifts from “protective refolding” to “promoting degradation” [41,42]. Following protein recognition, the ubiquitination cascade is initiated, with UBE2D1 playing a pivotal role as the core executor (E2 enzyme) in this process [43]. The primary function of RAD23 homolog A (RAD23A) is to serve as a key adaptor protein in the ubiquitin-proteasome system, establishing a bridging connection between the labeled proteins (ubiquitinated substrates) and the degradation machinery (proteasome) [44]. This protein facilitates the delivery of target proteins to the 20S proteasome core particle, and the 20S proteasome subunit alpha 2 (PSMA2), as a core component of this protein degradation machine [45,46], exhibits increased expression or activity that directly leads to excessive breakdown of muscle proteins (such as actin and myosin), thereby triggering muscle atrophy and functional decline. However, prior studies have not addressed the coordinated regulation of these genes in the context of T2DM, nor their interaction with inflammatory signaling. Our findings reveal that in T2DS, UBE2D1, PSMA2, and RAD23A are synergistically upregulated in response to TNFRSF1B-mediated inflammation and oxidative stress, forming a functional module that accelerates muscle protein degradation. This coordinated regulation of the ubiquitin-proteasome system by T2DS-specific stressors represents a novel mechanism and offers a new therapeutic perspective for T2DS.

Although our study advances our understanding of the pathogenesis of T2DS, several limitations should be noted. First, the scRNA-seq and bulk RNA-seq data were derived from public databases, and functional validation was limited to bioinformatics analyses; future in vitro and in vivo experiments (e.g., gene silencing or overexpression in myotubes or T2DS animal models) are needed to confirm the causal roles of the hub genes. Second, despite the performance of cross-validation, there remains the drawback of potential overfitting in the 127 combined machine learning models based on 11 algorithms. Third, we focused on the ubiquitin-proteasome pathway, but other pathways such as autophagy or mitochondrial dysfunction may also contribute to T2DS, which warrants further investigation. To validate these findings, future studies may employ several complementary strategies. First, quantitative real-time PCR (qPCR) and western blotting could be performed to confirm the differential expression of the seven hub genes in muscle biopsy samples from T2DM patients with and without sarcopenia. Second, in vitro functional studies using C2C12 myotubes under oxidative stress conditions induced by high glucose and palmitic acid could examine the effects of siRNA-mediated knockdown or overexpression of key hub genes on myotube diameter and ubiquitin-proteasome activity. Third, in vivo validation using a high-fat diet/streptozotocin-induced diabetic mouse model combined with hindlimb suspension-induced muscle atrophy could assess these hub genes in a physiologically relevant T2DS context, followed by histological analysis of myofiber cross-sectional area and immunohistochemical staining of the identified targets.

## 5. Conclusion

In conclusion, this study successfully identified seven core hub genes—TNFRSF1B, PSMA2, UBE2D1, UBE2N, HSP90AA1, RAD23A, and DNAJB1—intricately linking oxidative stress to the pathogenesis of T2DS. Through an integrated approach combining single-cell and bulk transcriptomics with advanced machine learning and PPI network analysis, we delineated a central mechanism where TNFRSF1B-mediated inflammatory signaling activates the ubiquitin-proteasome system, culminating in accelerated protein degradation and muscle atrophy. The consistent diagnostic performance of these genes across multiple datasets underscores their potential clinical relevance as robust biomarkers. These findings not only enhance our understanding of the molecular interplay between oxidative stress, inflammation, and proteostasis in T2DS but also pave the way for developing targeted diagnostic strategies and novel therapeutics aimed at interrupting this deleterious cycle, ultimately improving patient outcomes.

## Supporting information

S1 Fig Supplementary image of screening criteria for glmBoost+Enet (alpha = 0.6) model. A. AUC versus test set AUC scatter plot: deviation from diagonal indicates possible overfitting. B. Bar chart of AUC difference between training and testing models.
(PNG)

S1 Table A list of oxidative stress-related metabolic genes selected from the GeneCards database for subsequent analysis. Initially, 14130 genes were searched using the keyword ‘oxidative stress,’ and further screened using a Gene Inference Function Score (GIFtS) ≥ 10, resulting in 11931 genes.
(CSV)

S2 Table Feature genes selected by each machine learning algorithm combination across the 127 predictive models. For each algorithm combination (e.g., Lasso+Stepglm[both], SVM, Ridge, etc.), the table lists the genes that were retained after feature selection. This comprehensive screening served as the basis for identifying candidate hub genes, with the final model (Lasso+Stepglm[both]) highlighted in the first block. Only models with moderate gene numbers and consistent AUC performance were further considered to avoid overfitting.
(XLSX)
