# Supplementary figures and images for: Discovery of hub genes linking oxidative stress to type 2 diabetic sarcopenia using single-cell sequencing and machine learning
Source: PLoS One. 2026 Jul 7;21(7):e0352753. doi: 10.1371/journal.pone.0352753 (PMC13340795; doi:10.1371/journal.pone.0352753)

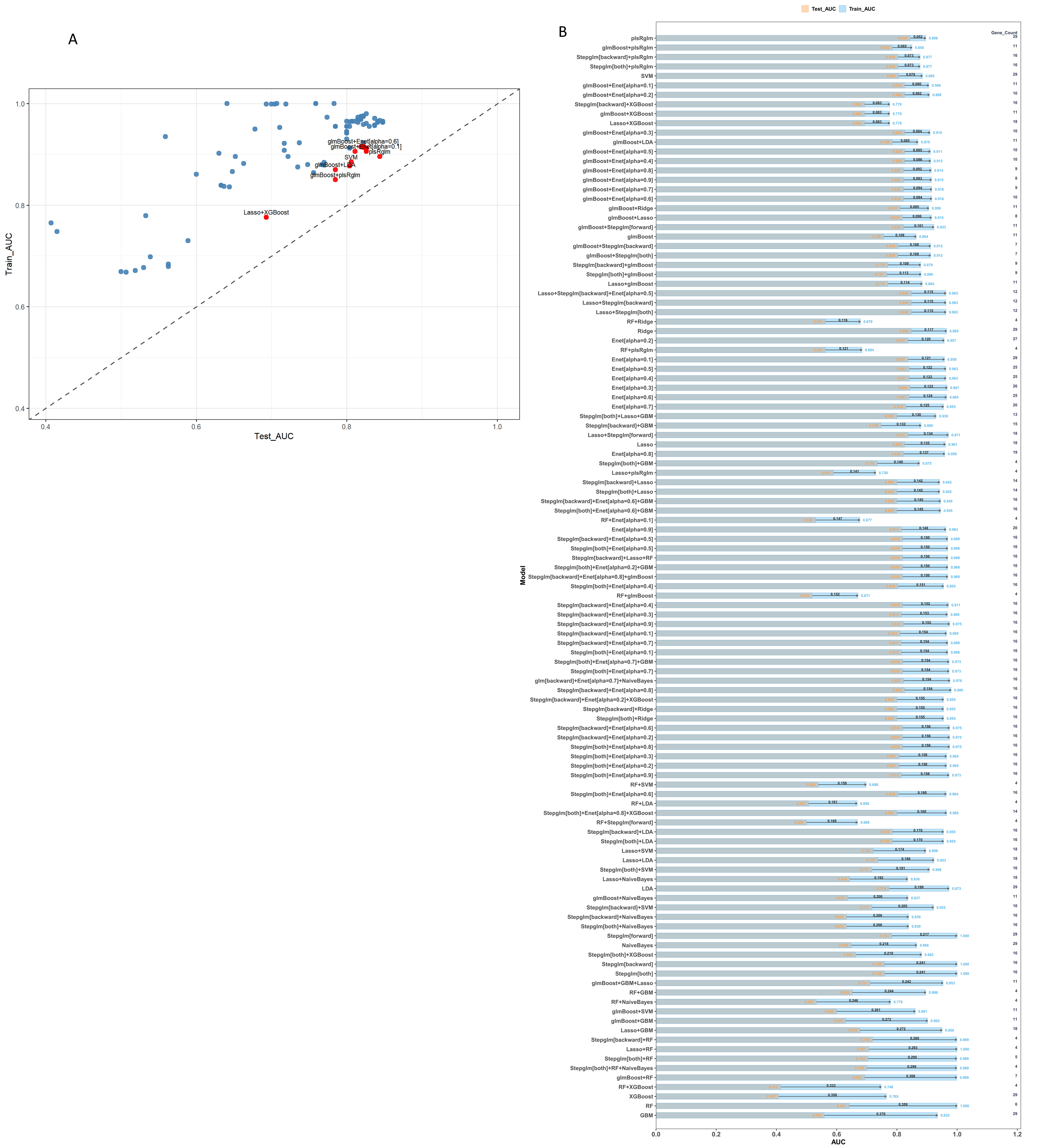

Supplement: S1 Fig — A. AUC versus test set AUC scatter plot: deviation from diagonal indicates possible overfitting. B. Bar chart of AUC difference between training and testing models. (PNG) [file pone.0352753.s001.png]
